# Vitamin D related genes in lung development and asthma pathogenesis

**DOI:** 10.1186/1755-8794-6-47

**Published:** 2013-11-05

**Authors:** Alvin T Kho, Sunita Sharma, Weiliang Qiu, Roger Gaedigk, Barbara Klanderman, Simin Niu, Chris Anderson, James S Leeder, Scott T Weiss, Kelan G Tantisira

**Affiliations:** 1 Children’s Hospital Informatics Program, Boston Children’s Hospital, 320 Longwood Avenue, Boston, MA 02115, USA; 2 Harvard Medical School, Boston, MA, USA; 3 Channing Division of Network Medicine, Brigham and Women’s Hospital, 181 Longwood Avenue, Boston, MA 02115, USA; 4 Pulmonary Division, Brigham and Women’s Hospital, Boston, MA, USA; 5 Children’s Mercy Hospital, Kansas City, MO, USA; 6 Partners Health Care Center for Personalized Genetic Medicine, Boston, MA, USA; 7 University of Rochester Medical Center, Rochester, NY, USA; 8 Channing Division of Network Medicine, Brigham and Women’s Hospital and Harvard Medical School, 181 Longwood Avenue, Boston, MA 02115, USA; 9 Division of Pediatric Clinical Pharmacology and Medical Toxicology, Children’s Mercy Hospital and Clinics, 2401 Gilham Road, Kansas City, MO 64108, USA; 10 Department of Microbiology and Immunology, University of Rochester Medical Center, 601 Elmwood Avenue, Rochester, NY 14642, USA

**Keywords:** Vitamin D, Cholecalciferol, Lung development, Asthma, Fetal programming

## Abstract

**Background:**

Poor maternal vitamin D intake is a risk factor for subsequent childhood asthma, suggesting that *in utero* changes related to vitamin D responsive genes might play a crucial role in later disease susceptibility. We hypothesized that vitamin D pathway genes are developmentally active in the fetal lung and that these developmental genes would be associated with asthma susceptibility and regulation in asthma.

**Methods:**

Vitamin D pathway genes were derived from PubMed and Gene Ontology surveys. Principal component analysis was used to identify characteristic lung development genes.

**Results:**

Vitamin D regulated genes were markedly over-represented in normal human (odds ratio OR 2.15, 95% confidence interval CI: 1.69-2.74) and mouse (OR 2.68, 95% CI: 2.12-3.39) developing lung transcriptomes. 38 vitamin D pathway genes were in both developing lung transcriptomes with >63% of genes more highly expressed in the later than earlier stages of development. In immortalized B-cells derived from 95 asthmatics and their unaffected siblings, 12 of the 38 (31.6%) vitamin D pathway lung development genes were significantly differentially expressed (OR 3.00, 95% CI: 1.43-6.21), whereas 11 (29%) genes were significantly differentially expressed in 43 control versus vitamin D treated immortalized B-cells from Childhood Asthma Management Program subjects (OR 2.62, 95% CI: 1.22-5.50). 4 genes, *LAMP3*, *PIP5K1B*, *SCARB2* and *TXNIP* were identified in both groups; each displays significant biologic plausibility for a role in asthma.

**Conclusions:**

Our findings demonstrate a significant association between early lung development and asthma–related phenotypes for vitamin D pathway genes, supporting a genomic mechanistic basis for the epidemiologic observations relating maternal vitamin D intake and childhood asthma susceptibility.

## Background

The prevalence of asthma, a disease affecting 300 million individuals worldwide [1], has risen substantially over the past 30 years. From an epidemiologic perspective, the increase in asthma has been greatest in industrialized countries and in those countries undergoing rapid urbanization [2,3]. This has led to the so-called Western lifestyles hypothesis, which states that factors accompanying the transition from a predominantly rural to a predominantly urban lifestyle may increase susceptibility to asthma and other auto-immune diseases [2-5]. One such factor is vitamin D deficiency. It has been estimated that one billion individuals worldwide are insufficient or deficient in vitamin D [6]. In Westernized countries, vitamin D levels tend to be low due to both increased urban (indoor) lifestyles and high use of sunscreen.

Multiple studies have now demonstrated an association between lower maternal vitamin D level during pregnancy and subsequent increased risk of childhood wheezing or asthma. For example, in an analysis of 1,194 mother-child pairs from Boston, MA, 3-year-old children born to mothers with vitamin D intake in the highest quartile during pregnancy had a 62% reduction in risk of recurrent wheeze (adjusted odds ratio OR 0.38, 95% confidence interval CI: 0.22-0.65) [7]. Similar analyses of 1,212 and 763 mother-child pairs from Scotland and Japan showed a 67% and 36% reduction in risk for subsequent persistent wheeze for children with maternal vitamin D intake in the highest quintile [8,9]. Overall, high maternal dietary vitamin D intake during pregnancy appears to be protective for the development of wheezing outcomes (OR 0.56, 95% CI: 0.42-0.73) [10].

The fetal origins or Barker [11] hypothesis states that *in utero* exposure to the maternal environment, including diet, may influence the eventual development of chronic disease. Globally, vitamin D has been postulated to be important in early pregnancy, regulating key target genes associated with implantation and implantation tolerance [12]. Vitamin D also regulates genes involved in the inflammation, immunity, cellular proliferation, and apoptosis associated with obstructive airways disease [13], likely via an epigenetic mechanism. Given the potential role that vitamin D plays in pregnancy, as well as the influence of maternal diet on subsequent childhood respiratory outcomes, it has been postulated that vitamin D deficiency directly affects programming within the developing fetal lung in a manner that may influence disease susceptibility [14,15]. We hypothesized that vitamin D pathway genes are transcriptionally active and temporally regulated during normal fetal lung development. Given the association of maternal vitamin D intake to subsequent childhood asthma, we further hypothesized that a significant subset of vitamin D genes important to normal fetal lung development would also be asthma susceptibility genes. We tested this hypothesis through an integrative analysis of developing mouse and human fetal lung transcriptomes. We identified key vitamin D pathway lung development genes and tested their transcriptomic association with asthma susceptibility.

## Methods

### Derivation of the vitamin D related gene set (VDRGS)

We assembled genes associated with vitamin D using both supervised and unsupervised approaches. In the supervised approach, we used genes recorded to be associated with vitamin D structure, function, regulation and signaling in Gene Ontology (GO, http://www.geneontology.org/ version May 2013) [16] or Entrez Gene (http://www.ncbi.nlm.nih.gov/gene version May 2013) databases. In the unsupervised approach, we used 212 unique human (195 homologous mouse) genes reported to be differentially regulated post vitamin D stimulation in human lymphoblastoid B cell lines [17].

### Microarray data

We used 3 developing lung time series datasets. The first is an expansion of the National Center for Biotechnology Information Gene Expression Omnibus (GEO, http://www.ncbi.nlm.nih.gov/geo/) GSE11539 [18] of C57BL6 mouse whole lung at embryonic days 9.5 (E9.5), 12.5, 14.5, 16.5, 18.5, and postnatal days 0 (P0), 2, 4, 7, 11, 13, 18, 24, 30, 56 in biological duplicates profiled on Affymetrix Mouse Gene 1.0 ST array courtesy of Carol J. Bult of the Jackson Laboratory, Bar Harbor, ME. The study protocol was approved by the Jackson Laboratory Animal Care and Use Committee #01011. The second GSE14334 consists of 38 human fetal lung samples from 53 to 154 estimated days post conception (dpc) profiled on the Affymetrix Human Genome U133 Plus 2.0 array [19]. The third GSE20954 consists of developing mouse whole lungs from E12 to P30 in duplicates profiled on Affymetrix Mouse Genome 430 Plus 2.0 array [20].

For asthma and vitamin D transcriptomic association analyses, we used 3 datasets: First, GSE8052 [21] consists of Epstein–Barr virus (EBV) transformed lymphoblastoid B-cells (LCLs) from a pediatric asthma family association study on the Affymetrix Human Genome U133 Plus 2.0 array. We restricted the data to 95 unaffected-affected sibling pairs (see Additional file 1: Table S1 and Figure S1). Second, we used LCLs from 43 asthmatic subjects from an ancillary genetics study of the Childhood Asthma Management Program (CAMP) [22,23] that was approved by the Brigham and Women’s Hospital Institutional Review Board # 1999-P-001549. All subjects or their legal guardians provided written informed consent to participate in the study protocols. LCLs were cultured in RPMI-1640 supplemented with 5% FBS and 1X Penicillin/Streptomycin/L-Glutamineto an average density of 200,000 cells/ml. The cells were then split and stimulated with sham (control) versus 1 μM 1,25-OH vitamin D for 72 hours. Total RNA was extracted from the cells using the Absolutely RNA Miniprep column purification system (Stratagene, LaJolla, CA) per manufacturer's instructions. Paired RNA samples were profiled on the Illumina HumanHT-12 V4 array. Third, GSE5145 consists of vitamin D versus sham stimulated normal human bronchial smooth muscle cells [24].

For each developmental dataset and the first asthma dataset, sample files were processed using the Robust Multi-array Analysis (RMA) quantile normalization [25] package in BioConductor (http://www.bioconductor.org/) to produce an N × M data matrix of RMA signals in the logarithmic base 2 (log2) scale. N is the total number of probe sets (genes) in the microarray platform and M is the total number of samples. The second asthma dataset was further processed using the probe-wise non-parametric regression function *locfit* in BioConductor [26] to minimize the effects of subject or age estimation–related variation in expression measurement and to model global gene-specific developmental expression patterns. For each probe set in a developmental dataset, we computed the linear correlation between replicate time series expression profiles to assess the reproducibility of its sample expression profile. When a gene (its Entrez Gene ID) is represented by >1 probe sets, we selected the probe set with the maximum linear correlation between replicate to be its unique representative.

In the asthma datasets, Wilcoxon signed rank test was used to determine differential expression between the paired samples, i.e., affected versus unaffected sibling, pre- versus post- vitamin D per subject at p < 0.05 significance threshold. When a gene (its Entrez Gene ID) is represented by >1 probe sets, we selected the probe set with the smallest signed rank p value as its unique representative.

### Principal component analysis for identifying characteristic genes in a developing organ transcriptome

Each developmental dataset is a N genes × M samples data matrix of RMA signals. First, the columns of the data matrix were standardized to average 0 and variance 1 since we were investigating sample variation in the sense of linear correlation, as opposed to Euclidean distance. Second, we performed principal component analysis (PCA) of sample time points in gene space, and obtained the loading coefficients for each gene in principal components 1 to 3 (PC1–3) [19,27-29]. Each principal component (PC) is a linear combination of N genes. The magnitude of the loading coefficient of a gene in a given PC corresponds to its contribution to the sample variation along that PC. For each PC, we ranked the genes in decreasing order of its loading coefficient magnitude. For example, the mouse gene H2-Ab1 ranked 42 in PC2 in the developing mouse lung dataset has the 42-nd highest loading coefficient magnitude in PC2. We define the characteristic genes for a PC to be the top 5% ranking genes in that PC. A gene that is characteristic in any one of the PC1–3 in a given dataset is defined to be a characteristic gene for that dataset.

### Fold change, bio-ontology enrichment and overlap analyses

The microarray reported gene expression intensity is an RMA signal in log2 scale. Suppose that each sample in a microarray dataset has a mutually exclusive condition label – A or B. The log2 fold change of a gene in A relative to B is the arithmetic average in A minus the arithmetic average in B. Fisher exact test on DAVID 6.7 http://david.abcc.ncifcrf.gov/[30] was used to determine specific bio-ontological enrichment in a given gene set relative to a background gene set at p < 0.05 significance threshold. More generally, two-sided Fisher exact test was used to assess the significance of overlaps between any pair of gene sets, and a 95% confidence interval (CI) was supplied for the odds ratio (OR).

## Results

### The vitamin D related gene set (VDRGS)

**Figure 1:**
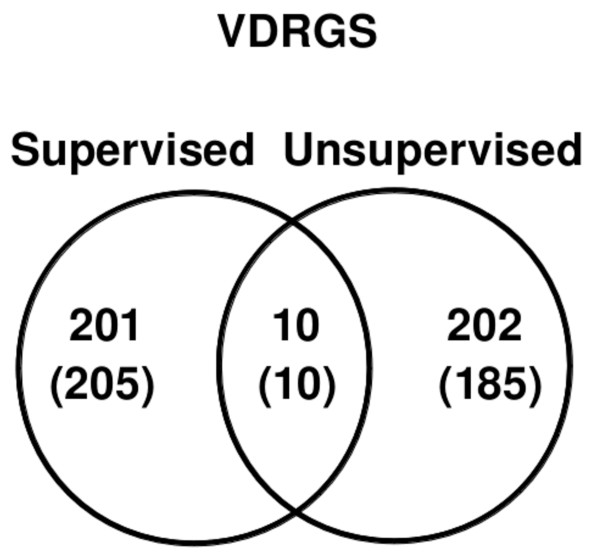
**Composition of the vitamin D related gene set (VDRGS).** Venn diagram of vitamin D related human gene sets assembled using supervised (GO and Entrez Gene databases) and unsupervised [17] approaches. Bracketed numbers (#) refer to number of homologous mouse genes.

For the supervised analysis, from GO, we found 24 GO terms that contain character strings “vitamin D*” or “cholecalciferol*”, where * denotes a wildcard character string. For example, the GO term “GO:0070561 vitamin D receptor signaling pathway” contains 4 unique human genes: *CYP24A1, CYP27B1, TRIM24* and *VDR*. The union of these 24 GO terms contained 67 unique human genes. Similarly, Entrez Gene had 201 unique human genes with a description field containing “vitamin D*” or “cholecalciferol*”. The supervised approach from GO and Entrez Gene combined yielded 211 unique human (215 homologous mouse) genes, see Figure 1 and Additional file 1: Table S1. In the unsupervised approach, as noted, we assembled 212 previously reported unique human (195 homologous mouse) genes resulting from differential expression following vitamin D stimulation [17], see Figure 1 and Additional file 1: Table S1. 10 genes were common to the sets of genes derived using supervised and unsupervised approaches: *CAMP, CD274, CYP19A1, CYP24A1, DHCR7, LGMN, MED13, NFKBIA, TNFSF4* and *VDR*. Together, the supervised and unsupervised approaches yielded 413 unique human (400 homologous mouse) genes that we define to be the vitamin D related gene set (VDRGS) in this study (Additional file 1: Table S1).

### Derivation of developing lung characteristic gene sets (DLCGS)

Here we identify the main contributor genes to transcriptome scale sample variation in the developing whole fetal lung using principal component analysis (PCA) following our previous work [19,28], see Materials and Methods. We used 2 independent developing whole lung transcriptome time series: The C57BL6 mouse time series (E9.5 to P56) spanned the major histo-morphological stages of lung development: pseudoglandular, canalicular, saccular and alveolar, GSE11539 expanded [18]. The human time series (53 to 154 dpc) spanned the pseudoglandular and canalicular stages, GSE14334 [19].

For each time series, we applied PCA of sample time points in transcriptome space. In each case we observed that the sample configuration along the first three principal components (PC1-3) correlated with age, time-to-birth or a transition between histo-morphological stages of lung development. The loading coefficient magnitude of a gene in a given principal component is proportional to its contribution to the sample variation along that principal component, see Materials and Methods. Therefore we define the genes with the top 5% highest loading coefficient magnitude in any one of PC1-3 to be the developing lung characteristic gene set (DLCGS) for the particular time series. These DLCGS consisted of 2,472 (human) and 2,495 (mouse) genes respectively. We had previously shown that these DLCGS were enriched for ontological attributes associated with developmental processes in general and the developing lung structure and function in particular [19,28] suggesting the DLCGS qualitatively capture developing lung biology.

### Over-representation of vitamin D related genes (VDRGS) in developing lung characteristic gene sets (DLCGS)

**Figure 2:**
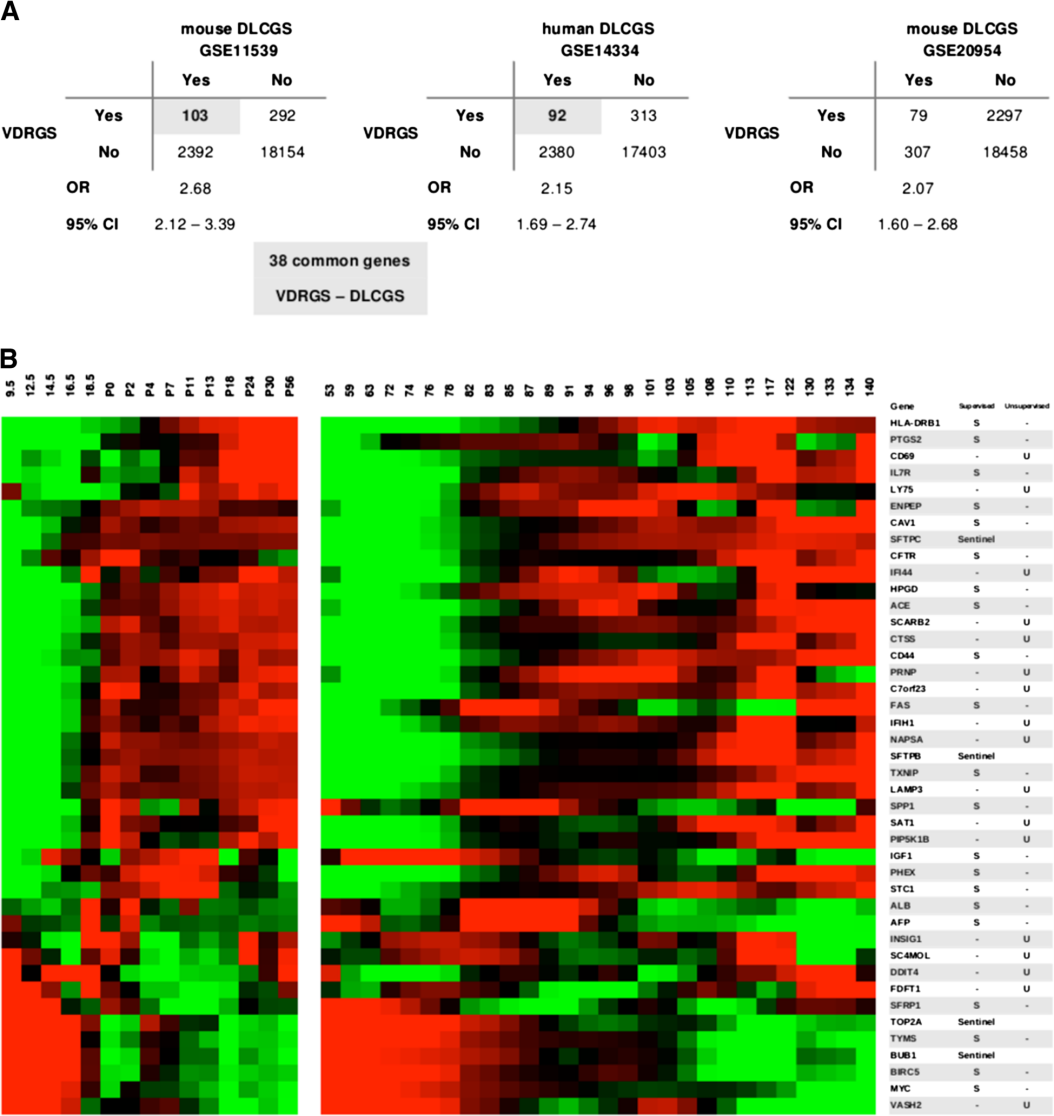
**Over-representation of vitamin D related genes in the developing lung transcriptome. A**: Contingency tables of overlaps between VDRGS and 3 independent developing lung characteristic gene sets (DLCGS). **B**: Heat maps of 38 VDRGS-DLCGS genes in the C57BL6 mouse (left) and human (right) developing lung time series. The expression signal of each gene in each time series has been standardized to average 0, variance 1 across their respective time intervals. Four sentinel genes – *BUB1*, *TOP2A*, *SFTPB* and *SFTPC* – included for visual reference.

We observed significant overlaps between the VDRGS with both DLCGS above: 103 VDRGS genes in the C57BL6 mouse DLCGS expanded GSE11439 (OR 2.68, 95% CI: 2.12-3.39), and 92 VDRGS genes in the human DLCGS GSE14334 (OR 2.15, 95% CI: 1.69-2.74) (Additional file 1: Figure S1). 38 VDRGS genes were common to both mouse and human DLCGS (Figure 2). The VDRGS was similarly found to overlap significantly with the DLCGS of an independent developing mouse lung time series (E12 to P30), GSE20954 (OR 2.07, 95% CI: 1.60-2.68) [20].

Of the 38 common VDRGS-DLCGS genes, 29 (76%) were expressed at a higher level in the later rather than earlier stages of the developing mouse lung; whereas 24 (63%) were expressed at a higher level in the later canalicular than earlier pseudoglandular stages of the developing human lung (Figure 2).

### Profile of 38 common VDRGS-DLCGS genes in independent pediatric asthma studies

Here we investigate the 38 VDRGS genes common to both the C57BL6 mouse and human DLCGS in independent studies of the pediatric lymphoblastoid B-cell (LCL) transcriptome in unaffected versus affected asthma sibling pairs [21] and vitamin D versus control treatment of LCLs from asthmatic subjects from the Childhood Asthma Management Program (CAMP) [31] (Table 1).

**Table 1:** 38 Vitamin D regulated lung developmental genes and their log2 fold changes in 3 asthma and vitamin D stimulation studies: Lymphoblastoid B-cells of pediatric asthma (GSE8052) and vitamin D treatment (CAMP 43), and normal human bronchial smooth muscle vitamin D treatment (GSE5145)

**Gene ID**	**Gene**	**Supervised**	**Unsupervised**	**GSE8052 asthma/unaffected**	**CAMP 43 vitamin D/control**	**GSE5145 vitamin D/control**
174	AFP	S	-	0.007	-0.047	0.094
213	ALB	S	-	-0.023	0.074	-0.130
332	BIRC5	S	-	-0.133	-0.030	0.055
355	FAS	S	-	0.098	0.060	0.189
857	CAV1	S	-	-0.058	**-0.169**	0.141
950	**SCARB2**	-	U	**-0.341**	**-0.124**	-0.174
960	CD44	S	-	**0.155**	0.107	0.325
969	CD69	-	U	0.046	**0.239**	0.022
1080	CFTR	S	-	0.022	-0.103	0.184
1520	CTSS	-	U	0.053	0.026	0.301
1636	ACE	S	-	0.062	-0.148	-0.262
2028	ENPEP	S	-	0.025	0.047	0.651
2222	FDFT1	-	U	0.080	0.056	0.125
3123	HLA-DRB1	S	-	**0.130**	-0.090	-0.107
3248	HPGD	S	-	0.022	-0.040	-0.134
3479	IGF1	S	-	**-0.094**	0.071	0.436
3575	IL7R	S	-	0.027	0.075	0.310
3638	INSIG1	-	U	-0.025	0.065	**1.223**
4065	LY75	-	U	**0.208**	0.108	-0.163
4609	MYC	S	-	**0.124**	**0.124**	-0.239
5251	PHEX	S	-	0.005	0.031	-0.084
5621	PRNP	-	U	0.036	**0.101**	0.362
0.362	PTGS2	S	-	0.013	0.087	0.044
6303	SAT1	-	U	0.077	**0.141**	0.064
6307	SC4MOL	-	U	0.070	0.058	0.297
6422	SFRP1	S	-	**0.084**	0.054	-0.335
6696	SPP1	S	-	-0.002	-0.073	-0.035
6781	STC1	S	-	-0.052	-0.085	-0.401
7298	TYMS	S	-	**-0.062**	0.032	-0.115
8395	**PIP5K1B**	-	U	**-0.239**	**0.149**	0.107
9476	NAPSA	-	U	-0.032	**0.388**	0.036
10561	IFI44	-	U	-0.183	**-0.098**	-0.326
10628	**TXNIP**	S	-	**-0.211**	**-0.360**	-0.151
27074	**LAMP3**	-	U	**0.127**	**0.080**	0.097
54541	DDIT4	-	U	0.080	-0.018	0.456
64135	IFIH1	-	U	**0.178**	0.058	-0.243
79161	TMEM243	-	U	0.000	0.054	0.168
79805	VASH2	-	U	0.090	**0.302**	0.179

Bold log2 values indicate statistical significance.

**Figure 3:**
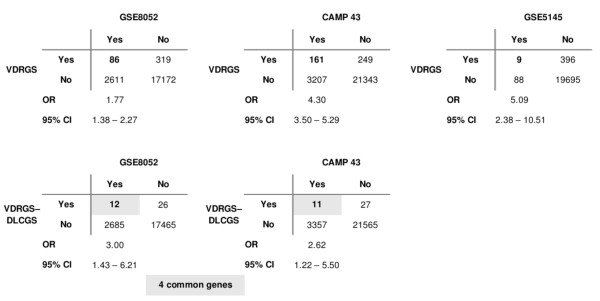
**Over-representation of vitamin D genes in 3 asthma and vitamin D stimulation studies.** Contingency tables of overlaps between VDRGS (and 38 VDRGS-DLCGS) and significantly differentially expressed genes in lymphoblastoid B-cells of pediatric asthma (GSE8052) and vitamin D treatment (CAMP 43), and normal human bronchial smooth muscle vitamin D treatment (GSE5145) studies.

For the pediatric asthma sibling pair study [21], 2,697 genes (of 20,188 unique genes measured) were differentially expressed between LCLs of 95 affected versus unaffected siblings pairs at p < 0.05. There was a significant overlap of 86 genes between this 2,697-gene set and the VDRGS (OR 1.77, 95% CI: 1.38-2.27). Focusing on the 38 common VDRGS-DLCGS genes, 12 (31%) were significantly differentially expressed between unaffected-affected siblings: CD44, HLA-DRB1, IFIH1, IGF1*, LAMP3, LY75, MYC, PIP5K1B*, SCARB2*, SFRP1, TXNIP* and TYMS*, where* indicates under expression in the affected relative to the unaffected sibling pair (OR 3.00, 95% CI: 1.43-6.21) (Figure 3).

For the vitamin D treatment study of 43 asthmatic CAMP subjects, 3,368 genes (of 24,960 unique genes measured) were differentially expressed between LCLs of vitamin D versus control treatment at p < 0.05. There was a significant overlap of 161 genes between this 3,368-gene set and the VDRGS (OR 4.30, 95% CI: 3.50-5.29) (Figure 3). Focusing on the 38 common VDRGS-DLCGS genes, 11 (22%) were significantly differentially expressed between control-vitamin D treatment: CAV1*, CD69, IFI44*, LAMP3, NAPSA, PIP5K1B, PRNP, SAT1, SCARB2*, TXNIP* and *VASH2,* where* indicates under-expression in vitamin D relative to control treatment (OR 2.62, 95% CI: 1.22-5.50) (Table 1). Four VDRGS-DLCGS genes were common to both asthma/VDRGS-DLCGS analyses above: *LAMP3, PIP5K1B2, SCARB2* and *TXNIP*. Interestingly, the SNP rs975645 of *PIP5K1B2* was found to be associated with asthma under a dominant genetic model in our previous CAMP study (p = 0.004) [32].

In an unrelated study of vitamin D stimulated normal human bronchial smooth muscle cells GSE5145 [24], of the 405 genes that were 2-fold differentially expressed between vitamin D versus control treatment, we found a significant overlap of 9 genes with the VDRGS (OR 5.09, 95% CI: 2.38-10.51) (Figure 3). Of these 9 genes, *INSIG1* was also in VDRGS-DLCGS.

## Discussion

We have performed a multi-staged analysis that demonstrates the prominence of vitamin D within the developing lung transcriptome and that supports the role of these developmental genes in asthma pathogenesis. Specifically, through interrogation of both supervised (literature based) and unsupervised (ChIP-Seq based) lists of vitamin D pathway genes, we have demonstrated that a significant proportion of vitamin D response elements are transcriptomically active during both normal human and mouse lung development. These vitamin D genes generally increase in expression throughout fetal lung development, with peak expression just prior to birth. We subsequently have shown that approximately 1/3 (12 of 38) of the vitamin D genes that jointly influence both human and murine lung development are also differentially expressed in LCLs derived from asthmatic children as compared to non-asthmatic sibling controls. Overall, vitamin D lung developmental genes were three times as likely to be differentially expressed in these asthmatics, when compared to non-vitamin D genes. Finally, we noted that, of the 12 vitamin D lung developmental genes transcriptomically related to asthma susceptibility, 4 – *LAMP1, PIP5K1B, SCARB2*, and *TXNIP* – were significantly differentially expressed upon administration of vitamin D to cells derived from asthmatic children. Thus, multiple common vitamin D response elements appear to be important in both the developing lung and asthma, thereby providing a genomic rationale as a basis for the influence of maternal diet on later asthma susceptibility.

This work lends credence to the evolving literature surrounding the developmental origins hypothesis of complex disease pathogenesis. Prior work have established that dietary changes, including protein and caloric restriction, as well as environmental exposures in pregnant animals can result in changes in gene expression in the neonate that persist into adulthood [33-36]. Moreover, in humans, maternal vitamin D levels affect both placental calcium transport and bone mass in later life. These effects are thought to be mediated by vitamin D induction of the *PMCA* (plasma membrane Ca^2+^ ATPase) gene [8,37]. While the current study was not designed to demonstrate a direct correlation between maternal vitamin D status and neonatal outcomes, it does clearly indicate that vitamin D regulated processes are a part of the normal fetal lung developmental process. Combining this with the strong epidemiologic evidence linking maternal vitamin D status during pregnancy to subsequent asthma, our data suggest that maternal vitamin D insufficiency may lead to differential developmental regulation of key vitamin D genes within the fetal lung and thus, increase risk of childhood asthma. This hypothesis is further supported by the differences in expression of a significant number of developmental vitamin D genes in siblings discordant for asthma.

Our data further suggest that a subset of the lung developmental vitamin D asthma genes continue to be actively regulated in later life by vitamin D, and thus, may continue to modify the asthma phenotype. The 4 genes that demonstrate this phenomenon in immortalized B cells derived directly from persistent childhood asthmatics likely represent a subset of vitamin D pathway genes involved in the immune mediation of asthma. Given their fetal lung and subsequent immune cell localization, it is possible that the remainder of the developmental 12 vitamin D genes that are differentially expressed in asthma may influence asthma in cells specific to the lung, such as epithelial or airway smooth muscle cells.

As mentioned, 4 of the developmental genes: *LAMP1, PIP5K1B, SCARB2* and *TXNIP*, were differentially expressed in both asthma and upon stimulation of immortalized B-cells derived from asthmatics, suggesting a possible role of these genes in modulating the immune response in asthma. Interestingly, although these genes have generally not been implicated in allergic airways disease, each of these genes may be related to asthma pathogenesis via a distinct molecular mechanism. *TXNIP*, also known as vitamin D3 up-regulated protein 1, is required for the development of natural killer cells [38]. In turn, CD4 + Vα24+ natural killer cells are significantly decreased in association with infection-associated asthma exacerbations and sputum eosinophil counts [39]. Notably, our microarray association demonstrated decreased expression of *TXNIP* in asthma; this association has been independently validated in a comparison of active asthmatics vs. normal volunteers [40].

While the other three genes have not been associated with asthma, they provide potentially interesting insights into the diversity of vitamin D biology. *LAMP3* appears to be a marker of dendritic cell maturation [41] and has been implicated in the pathogenesis of psoriasis vulgaris [42]. *PIP5K1B* regulates calcium signaling in mast cells[43]. Both the dendritic cell and the mast cell are key regulators in the initiation of the asthmatic inflammatory response. Furthermore, the SNP rs975645 of *PIP5K1B2* was found to be associated with asthma under a dominant genetic model in our previous CAMP study (p = 0.004) [32]. In lieu of direct actions on inflammatory cells, *SCAR2B* appears to modulate the normal maturation of phagosomes and autophagosomes [44,45]. While the exact role of autophagy in asthma remains to be determined, it has been hypothesized that autophagy is likely an important modulator of the lack of viral-induced apoptosis noted in subjects with asthma [46]. Consistent with this, a recent brief report noted the presence of autophagosomes in the airways of a subject with asthma; a comparable tissue from a control subject failed to detect any autophagosomes [47].

There are several limitations to our data. Our human lung samples were obtained from healthy aborted fetuses and thus inherently limited in their gestational age range to ≤20 estimated weeks. While the vitamin D pathway appears to be active in early fetal development [12], it is unclear if perturbations in early or late gestation are most salient to asthma susceptibility. For this reason, we intersected our human developmental vitamin D list with a murine developmental dataset in which later developmental time periods were represented. Our sampling technique also precludes us from specifically ascertaining the direct effect of maternal vitamin D levels on the developing human lung. Instead, we focused on a combined approach that focused on the identification of significant vitamin D regulatory genes from the literature and a carefully performed ChIP-Seq study of the vitamin D receptor [17]. Finally, our asthma expression dataset analyses were performed in immortalized B-cells derived from asthmatics. While this may not specifically generalize the findings of the fetal lung expression datasets, the B-cell is a relevant asthma target cell [48-50] supporting the validity of these analyses. Only a small number of genes are specifically affected by the immortalization process [51]; none of these were among our reported vitamin D genes. Moreover, we have been able to previously correlate expression response of these genes to clinical asthma outcomes [31], further supporting the validity of our approach.

## Conclusions

In conclusion, we have demonstrated that vitamin D genes are actively regulated in the developing human fetal lung and that a disproportionate number of these genes are differentially regulated in asthma. Not only does our study provide a mechanistic basis that helps to explain the developmental associations of vitamin D with asthma, it may also provide a methodologic blueprint for rationally interrogating diverse developmental pathways for their genomic association with subsequent disease outcomes.

## Abbreviations

ChIP-Seq: Chromatin immunoprecipation with massively parallel DNA sequencing; DLCGS: Developing lung characteristic gene set; GO: Gene Ontology; ID: Identifier; Log2: Logarithm base 2; PC#: #-th principal component; PCA: Principal component analysis; RMA: Robust multi-array analysis; VDRGS: Vitamin D related gene set.

## Competing interests

The authors declare that they have no competing interests.

## Authors’ contributions

ATK contributed to the design, data analysis and writing. SS contributed to the data collection, analysis and writing. WQ, RG, BK, SM, CA, JSL contributed to the data collection and writing. STW contributed to the design and writing. KTG contributed to the design, analysis and writing. All authors read and approved the final manuscript.

## Pre-publication history

The pre-publication history for this paper can be accessed here:

http://www.biomedcentral.com/1755-8794/6/47/prepub

## Supplementary Material

Additional file 1: Table S1 413 vitamin D related genes. **Figure S1.** Heat maps of 103 and 92 vitamin D related lung genes overlapping with the developing lung characteristic genes of C57BL6 mouse (A) and human (B) developing lung time series respectively. The expression signal of each gene in each time series has been standardized to average 0, variance 1 across their respective time intervals. Four sentinel genes – *BUB1, TOP2A, SFTPB* and *SFTPC* – included for visual reference. Click here for file
