# Supplementary material for: Vitamin D related genes in lung development and asthma pathogenesis
Source: BMC Med Genomics. 2013 Nov 5;6:47. doi: 10.1186/1755-8794-6-47 (PMC4228235; doi:10.1186/1755-8794-6-47)

**Vitamin D related genes in lung development and asthma pathogenesis.**

**Additional / Supplemental Data Files**

**Table S1**. 413 vitamin D related genes.

| **Homologene ID** | **# human genes** | **Human Entrez Gene ID** | **Human Gene Symbol** | **# mouse genes** | **Mouse Entrez Gene ID** | **Mouse Gene Symbol** | **Supervised** | **Unsupervised** |
| --- | --- | --- | --- | --- | --- | --- | --- | --- |
| 55496 | 1 | 5243 | ABCB1 | 1 | 18671 | Abcb1a | S | - |
| 55873 | 1 | 225 | ABCD2 | 1 | 26874 | Abcd2 | - | U |
| 37351 | 1 | 1636 | ACE | 1 | 11421 | Ace | S | - |
| 37561 | 1 | 2180 | ACSL1 | 1 | 14081 | Acsl1 | - | U |
| 3278 | 1 | 2181 | ACSL3 | 1 | 74205 | Acsl3 | - | U |
| 56282 | 1 | 2182 | ACSL4 | 1 | 50790 | Acsl4 | - | U |
| 2395 | 1 | 6868 | ADAM17 | 1 | 11491 | Adam17 | S | - |
| 5865 | 1 | 137872 | ADHFE1 | 1 | 76187 | Adhfe1 | - | U |
| 881 | 1 | 173 | AFM | 1 | 280662 | Afm | S | - |
| 36278 | 1 | 174 | AFP | 1 | 11576 | Afp | S | - |
| 83226 | 1 | 9447 | AIM2 | 1 | 383619 | Aim2 | - | U |
| 3785 | 1 | 207 | AKT1 | 1 | 11651 | Akt1 | S | - |
| 405 | 1 | 213 | ALB | 1 | 11657 | Alb | S | - |
| 561 | 1 | 240 | ALOX5 | 1 | 11689 | Alox5 | S | - |
| 37314 | 1 | 249 | ALPL | 1 | 11647 | Alpl | S | - |
| 68060 | 1 | 268 | AMH | 1 | 11705 | Amh | S | - |
| 20857 | 1 | 302 | ANXA2 | 1 | 12306 | Anxa2 | S | - |
| 14695 | 1 | 221656 | AOF1 | 1 | 218214 | Aof1 | - | U |
| 47900 | 1 | 335 | APOA1 | 1 | 11806 | Apoa1 | S | - |
| 21025 | 1 | 360 | AQP3 | 1 | 11828 | Aqp3 | S | - |
| 28 | 1 | 367 | AR | 1 | 11835 | Ar | S | - |
| 3468 | 1 | 9181 | ARHGEF2 | 1 | 16800 | Arhgef2 | - | U |
| 12847 | 1 | 51742 | ARID4B | 1 | 94246 | Arid4b | - | U |
| 41692 | 1 | 554235 | ASPDH | 1 | 68352 | Aspdh | S | - |
| 3190 | 1 | 573 | BAG1 | 1 | 12017 | Bag1 | S | - |
| 22651 | 1 | 9031 | BAZ1B | 1 | 22385 | Baz1b | S | - |
| 2794 | 1 | 8678 | BECN1 | 1 | 56208 | Becn1 | S | - |
| 104130 | 1 | 632 | BGLAP | 3 | 12096 // 12097 // 12095 | Bglap // Bglap2 // Bglap-rs1 | S | - |
| 37450 | 1 | 332 | BIRC5 | 1 | 11799 | Birc5 | S | - |
| 926 | 1 | 650 | BMP2 | 1 | 12156 | Bmp2 | S | - |
| 7247 | 1 | 652 | BMP4 | 1 | 12159 | Bmp4 | S | - |
| 1300 | 1 | 654 | BMP6 | 1 | 12161 | Bmp6 | S | - |
| 20410 | 1 | 655 | BMP7 | 1 | 12162 | Bmp7 | S | - |
| 2990 | 1 | 664 | BNIP3 | 1 | 100042570 | LOC100042570 | - | U |
| 3195 | 1 | 665 | BNIP3L | 2 | 12177 | Bnip3l | - | U |
| 9781 | 1 | 54836 | BSPRY | 1 | 192120 | Bspry | S | - |
| 52233 | 1 | 151888 | BTLA | 1 | 208154 | Btla | - | U |
| 19024 | 1 | 122525 | C14orf28 | 1 | 217648 | Gm527 | - | U |
| 10993 | 1 | 29071 | C1GALT1C1 | 1 | 59048 | C1galt1c1 | - | U |
| 21009 | 1 | 9473 | C1orf38 | 1 | 230787 | BC013712 | - | U |
| 20413 | 1 | 728 | C5AR1 | 1 | 12273 | C5ar1 | S | - |
| 51384 | 1 | 115004 | C6orf150 | 1 | 214763 | E330016A19Rik | - | U |
| 41561 | 1 | 79161 | C7orf23 | 1 | 652925 | 4930420K17Rik | - | U |
| 11933 | 1 | 138241 | C9orf85 | 1 | 66206 | 1110059E24Rik | - | U |
| 69212 | 1 | 51719 | CAB39 | 1 | 12283 | Cab39 | - | U |
| 21026 | 1 | 793 | CALB1 | 1 | 12307 | Calb1 | S | - |
| 110678 | 1 | 820 | CAMP | 1 | 12796 | Camp | S | U |
| 332 | 1 | 846 | CASR | 1 | 12374 | Casr | S | - |
| 7658 | 1 | 831 | CAST | 1 | 12380 | Cast | - | U |
| 1330 | 1 | 857 | CAV1 | 1 | 12389 | Cav1 | S | - |
| 37524 | 1 | 873 | CBR1 | 1 | 12408 | Cbr1 | S | - |
| 20332 | 1 | 874 | CBR3 | 1 | 109857 | Cbr3 | S | - |
| 7529 | 1 | 6367 | CCL22 | 1 | 20299 | Ccl22 | - | U |
| 48153 | 1 | 6351 | CCL4 | 1 | 20303 | Ccl4 | - | U |
| 47975 | 1 | 9560 | CCL4L1 | 0 | - | - | - | U |
| 47975 | 1 | 388372 | CCL4L2 | 0 | - | - | - | U |
| 4344 | 1 | 4345 | CD200 | 1 | 17470 | Cd200 | - | U |
| 8560 | 1 | 29126 | CD274 | 1 | 60533 | Cd274 | S | U |
| 7663 | 1 | 23607 | CD2AP | 1 | 12488 | Cd2ap | - | U |
| 1345 | 1 | 952 | CD38 | 1 | 12494 | Cd38 | - | U |
| 508 | 1 | 960 | CD44 | 1 | 12505 | Cd44 | S | - |
| 1346 | 1 | 961 | CD47 | 1 | 16423 | Cd47 | - | U |
| 90963 | 1 | 969 | CD69 | 1 | 12515 | Cd69 | - | U |
| 31053 | 1 | 973 | CD79A | 1 | 12518 | Cd79a | - | U |
| 3804 | 1 | 941 | CD80 | 1 | 12519 | Cd80 | - | U |
| 10443 | 1 | 942 | CD86 | 1 | 12524 | Cd86 | - | U |
| 8050 | 1 | 976 | CD97 | 1 | 26364 | Cd97 | - | U |
| 10285 | 1 | 55536 | CDCA7L | 1 | 217946 | Cdca7l | - | U |
| 963 | 1 | 1021 | CDK6 | 1 | 12571 | Cdk6 | - | U |
| 49467 | 1 | 51265 | CDKL3 | 1 | 213084 | Cdkl3 | - | U |
| 2999 | 1 | 1027 | CDKN1B | 1 | 12576 | Cdkn1b | S | - |
| 36081 | 1 | 1032 | CDKN2D | 1 | 12581 | Cdkn2d | S | - |
| - | 1 | 374286 | CDRT1 | - | - | - | - | U |
| 968 | 1 | 1045 | CDX2 | 1 | 12591 | Cdx2 | S | - |
| 40574 | 1 | 23436 | CELA3B | 1 | 67868 | Cela3b | S | - |
| 7652 | 1 | 8837 | CFLAR | 1 | 12633 | Cflar | - | U |
| 55465 | 1 | 1080 | CFTR | 1 | 12638 | Cftr | S | - |
| 68174 | 1 | 1105 | CHD1 | 1 | 12648 | Chd1 | - | U |
| 19067 | 1 | 55636 | CHD7 | 1 | 320790 | Chd7 | - | U |
| 40809 | 1 | 9069 | CLDN12 | 1 | 64945 | Cldn12 | S | - |
| 9621 | 1 | 9075 | CLDN2 | 1 | 12738 | Cldn2 | S | - |
| 43154 | 1 | 54102 | CLIC6 | 1 | 209195 | Clic6 | - | U |
| 11683 | 1 | 79789 | CLMN | 1 | 94040 | Clmn | - | U |
| 8956 | 1 | 22866 | CNKSR2 | 1 | 245684 | Cnksr2 | S | - |
| 73874 | 1 | 1277 | COL1A1 | 1 | 12842 | Col1a1 | S | - |
| 74 | 1 | 1311 | COMP | 1 | 12845 | Comp | S | - |
| 3124 | 1 | 9318 | COPS2 | 1 | 12848 | Cops2 | S | - |
| 55611 | 1 | 1380 | CR2 | 1 | 12902 | Cr2 | - | U |
| 476 | 1 | 1401 | CRP | 1 | 12944 | Crp | S | - |
| 21280 | 1 | 10491 | CRTAP | 1 | 56693 | Crtap | - | U |
| 55615 | 1 | 1473 | CST5 | 1 | 58214 | Cst10 | S | - |
| 20867 | 1 | 1520 | CTSS | 1 | 13040 | Ctss | - | U |
| 1022 | 1 | 1522 | CTSZ | 1 | 64138 | Ctsz | - | U |
| 37434 | 1 | 8029 | CUBN | 1 | 65969 | Cubn | S | - |
| 20739 | 1 | 7852 | CXCR4 | 1 | 12767 | Cxcr4 | - | U |
| 37347 | 1 | 1583 | CYP11A1 | 1 | 13070 | Cyp11a1 | S | - |
| 30955 | 1 | 1588 | CYP19A1 | 1 | 13075 | Cyp19a1 | S | U |
| 68062 | 1 | 1543 | CYP1A1 | 1 | 13076 | Cyp1a1 | S | - |
| 68094 | 1 | 1591 | CYP24A1 | 1 | 13081 | Cyp24a1 | S | U |
| 37349 | 1 | 1592 | CYP26A1 | 1 | 13082 | Cyp26a1 | S | - |
| 23179 | 1 | 56603 | CYP26B1 | 1 | 232174 | Cyp26b1 | S | - |
| 28089 | 1 | 340665 | CYP26C1 | 1 | 546726 | Cyp26c1 | S | - |
| 36040 | 1 | 1593 | CYP27A1 | 1 | 104086 | Cyp27a1 | S | - |
| 37139 | 1 | 1594 | CYP27B1 | 1 | 13115 | Cyp27b1 | S | - |
| 73894 | 1 | 1555 | CYP2B6 | 1 | 13088 | Cyp2b10 | S | - |
| 110445 | 1 | 1559 | CYP2C9 | 0 | - | - | S | - |
| 75210 | 1 | 120227 | CYP2R1 | 1 | 244209 | Cyp2r1 | S | - |
| 111391 | 1 | 1576 | CYP3A4 | 5 | 100041375 // 13112 // 337924 // 13114 // 53973 | Cyp3a41b // Cyp3a11 // Cyp3a44 // Cyp3a16 // Cyp3a41a | S | - |
| 36635 | 1 | 23002 | DAAM1 | 1 | 208846 | Daam1 | - | U |
| 1035 | 1 | 1628 | DBP | 1 | 13170 | Dbp | S | - |
| 10400 | 1 | 54541 | DDIT4 | 1 | 74747 | Ddit4 | - | U |
| 52110 | 1 | 203522 | DDX26B | 1 | 236790 | Ddx26b | - | U |
| 52660 | 1 | 91351 | DDX60L | 0 | - | - | - | U |
| 122147 | 1 | 1673 | DEFB4 | 0 | - | - | S | - |
| 1042 | 1 | 1717 | DHCR7 | 1 | 13360 | Dhcr7 | S | U |
| 68396 | 1 | 1758 | DMP1 | 1 | 13406 | Dmp1 | S | - |
| 105716 | 1 | 667 | DST | 0 | - | - | S | - |
| 10633 | 1 | 56986 | DTWD1 | 1 | 69185 | Dtwd1 | - | U |
| 5215 | 1 | 11221 | DUSP10 | 1 | 63953 | Dusp10 | - | U |
| 1068 | 1 | 1889 | ECE1 | 1 | 230857 | Ece1 | - | U |
| 37822 | 1 | 8411 | EEA1 | 1 | 216238 | Eea1 | - | U |
| 11599 | 1 | 79631 | EFTUD1 | 1 | 101592 | Eftud1 | - | U |
| 11456 | 1 | 79071 | ELOVL6 | 1 | 170439 | Elovl6 | - | U |
| 68216 | 1 | 2028 | ENPEP | 1 | 13809 | Enpep | S | - |
| 122202 | 1 | 57089 | ENTPD7 | 1 | 93685 | Entpd7 | - | U |
| 12630 | 1 | 94240 | EPSTI1 | 1 | 108670 | Epsti1 | - | U |
| 47906 | 1 | 2099 | ESR1 | 1 | 13982 | Esr1 | S | - |
| 1100 | 1 | 2100 | ESR2 | 1 | 13983 | Esr2 | S | - |
| 3837 | 1 | 2113 | ETS1 | 1 | 23871 | Ets1 | S | - |
| 3276 | 1 | 2119 | ETV5 | 1 | 104156 | Etv5 | - | U |
| 22753 | 1 | 3992 | FADS1 | 1 | 76267 | Fads1 | - | U |
| 3149 | 1 | 9415 | FADS2 | 1 | 56473 | Fads2 | - | U |
| 57065 | 1 | 414918 | FAM116B | 1 | 69440 | Fam116b | - | U |
| 56783 | 1 | 54855 | FAM46C | 1 | 74645 | Fam46c | - | U |
| 105729 | 1 | 58516 | FAM60A | 1 | 56306 | Fam60a | - | U |
| 11564 | 1 | 79567 | FAM65A | 1 | 75687 | Fam65a | - | U |
| 69247 | 1 | 55711 | FAR2 | 1 | 330450 | Far2 | - | U |
| 27 | 1 | 355 | FAS | 1 | 14102 | Fas | S | - |
| 533 | 1 | 356 | FASLG | 1 | 14103 | Fasl | S | - |
| 117451 | 1 | 55294 | FBXW7 | 1 | 50754 | Fbxw7 | - | U |
| 3281 | 1 | 2222 | FDFT1 | 1 | 14137 | Fdft1 | - | U |
| 31216 | 1 | 2230 | FDX1 | 1 | 14148 | Fdx1 | S | - |
| 10771 | 1 | 8074 | FGF23 | 1 | 64654 | Fgf23 | S | - |
| 1533 | 1 | 2335 | FN1 | 1 | 14268 | Fn1 | S | - |
| 4846 | 1 | 10818 | FRS2 | 1 | 327826 | Frs2 | - | U |
| 20750 | 1 | 8321 | FZD1 | 1 | 14362 | Fzd1 | S | - |
| 20377 | 1 | 2535 | FZD2 | 1 | 57265 | Fzd2 | S | - |
| 48022 | 1 | 645051 | GAGE13 | 0 | - | - | - | U |
| 3297 | 1 | 2590 | GALNT2 | 1 | 108148 | Galnt2 | - | U |
| 486 | 1 | 2638 | GC | 1 | 14473 | Gc | S | - |
| 45639 | 1 | 9648 | GCC2 | 1 | 70297 | Gcc2 | - | U |
| 3490 | 1 | 9247 | GCM2 | 1 | 107889 | Gcm2 | S | - |
| 3854 | 1 | 2672 | GFI1 | 1 | 14581 | Gfi1 | S | - |
| - | 1 | 2688 | GH1 | 1 | 14599 | Gh | S | - |
| 47922 | 1 | 2720 | GLB1 | 1 | 12091 | Glb1 | - | U |
| 74720 | 1 | 152007 | GLIPR2 | 1 | 384009 | Glipr2 | - | U |
| 55976 | 1 | 10672 | GNA13 | 1 | 14674 | Gna13 | - | U |
| 1158 | 1 | 2959 | GTF2B | 1 | 229906 | Gtf2b | S | - |
| 21237 | 1 | 3001 | GZMA | 1 | 14938 | Gzma | S | - |
| 48250 | 1 | 8841 | HDAC3 | 1 | 15183 | Hdac3 | S | - |
| 503 | 1 | 3082 | HGF | 1 | 15234 | Hgf | S | - |
| 1171 | 1 | 3091 | HIF1A | 1 | 15251 | Hif1a | - | U |
| 68766 | 1 | 28996 | HIPK2 | 1 | 15258 | Hipk2 | - | U |
| 4900 | 1 | 3097 | HIVEP2 | 1 | 15273 | Hivep2 | - | U |
| 85991 | 1 | 3123 | HLA-DRB1 | 1 | 14969 | H2-Eb1 | S | - |
| 1609 | 1 | 3157 | HMGCS1 | 1 | 208715 | Hmgcs1 | - | U |
| 7365 | 1 | 3206 | HOXA10 | 1 | 15395 | Hoxa10 | S | - |
| 68095 | 1 | 3248 | HPGD | 1 | 15446 | Hpgd | S | - |
| 3774 | 1 | 55806 | HR | 1 | 15460 | Hr | S | - |
| 68464 | 1 | 3320 | HSP90AA1 | 1 | 15519 | Hsp90aa1 | S | - |
| 37590 | 1 | 3418 | IDH2 | 1 | 269951 | Idh2 | - | U |
| 4683 | 1 | 10561 | IFI44 | 1 | 99899 | Ifi44 | - | U |
| 48468 | 1 | 10964 | IFI44L | 1 | 15061 | H28 | - | U |
| 49720 | 1 | 2537 | IFI6 | 0 | - | - | - | U |
| 32535 | 1 | 64135 | IFIH1 | 1 | 71586 | Ifih1 | - | U |
| 78213 | 1 | 3434 | IFIT1 | 1 | 112419 | 2010002M12Rik | - | U |
| 74501 | 1 | 8519 | IFITM1 | 1 | 68713 | Ifitm1 | - | U |
| 1640 | 1 | 3456 | IFNB1 | 1 | 15977 | Ifnb1 | S | - |
| 55526 | 1 | 3458 | IFNG | 1 | 15978 | Ifng | S | - |
| 515 | 1 | 3479 | IGF1 | 1 | 16000 | Igf1 | S | - |
| 498 | 1 | 3484 | IGFBP1 | 1 | 16006 | Igfbp1 | S | - |
| 55948 | 1 | 10320 | IKZF1 | 1 | 22778 | Ikzf1 | - | U |
| 478 | 1 | 3586 | IL10 | 1 | 16153 | Il10 | S | - |
| 487 | 1 | 3600 | IL15 | 1 | 16168 | Il15 | S | - |
| 10287 | 1 | 55540 | IL17RB | 1 | 50905 | Il17rb | - | U |
| 480 | 1 | 3552 | IL1A | 1 | 16175 | Il1a | S | - |
| 481 | 1 | 3553 | IL1B | 1 | 16176 | Il1b | S | - |
| 360 | 1 | 3559 | IL2RA | 1 | 16184 | Il2ra | S | - |
| 502 | 1 | 3569 | IL6 | 1 | 16193 | Il6 | S | - |
| 1646 | 1 | 3575 | IL7R | 1 | 16197 | Il7r | S | - |
| 4047 | 1 | 3638 | INSIG1 | 1 | 231070 | Insig1 | - | U |
| 9400 | 1 | 51141 | INSIG2 | 1 | 72999 | Insig2 | - | U |
| 20090 | 1 | 3643 | INSR | 1 | 16337 | Insr | S | - |
| 76214 | 1 | 253430 | IPMK | 1 | 69718 | Ipmk | - | U |
| 5835 | 1 | 79711 | IPO4 | 1 | 75751 | Ipo4 | S | - |
| 82429 | 1 | 9922 | IQSEC1 | 1 | 232227 | Iqsec1 | - | U |
| 1207 | 1 | 3656 | IRAK2 | 1 | 108960 | Irak2 | - | U |
| 1629 | 1 | 3394 | IRF8 | 1 | 15900 | Irf8 | - | U |
| 38105 | 1 | 10265 | IRX5 | 1 | 54352 | Irx5 | S | - |
| 20091 | 1 | 3655 | ITGA6 | 1 | 16403 | Itga6 | S | - |
| 179 | 1 | 3691 | ITGB4 | 1 | 192897 | Itgb4 | S | - |
| 11186 | 1 | 81618 | ITM2C | 1 | 64294 | Itm2c | - | U |
| 86915 | 1 | 3713 | IVL | 0 | - | - | S | - |
| 7390 | 1 | 3726 | JUNB | 1 | 16477 | Junb | - | U |
| 20834 | 1 | 8850 | KAT2B | 2 | 18519 | Kat2b | - | U |
| 68242 | 1 | 3756 | KCNH1 | 1 | 16510 | Kcnh1 | S | - |
| 1696 | 1 | 3783 | KCNN4 | 1 | 16534 | Kcnn4 | - | U |
| 10196 | 1 | 55818 | KDM3A | 1 | 104263 | Kdm3a | - | U |
| 106645 | 1 | 9840 | KIAA0748 | 0 | - | - | - | U |
| 32778 | 1 | 84451 | KIAA1804 | 1 | 234878 | BC021891 | - | U |
| 8196 | 1 | 22944 | KIN | 1 | 16588 | Kin | - | U |
| 68415 | 1 | 9365 | KL | 1 | 16591 | Kl | S | - |
| 32288 | 1 | 51621 | KLF13 | 1 | 50794 | Klf13 | - | U |
| x | 1 | 354 | KLK3 | 0 | - | - | S | - |
| 75000 | 1 | 25818 | KLK5 | 1 | 68668 | Klk5 | S | - |
| 37998 | 1 | 5650 | KLK7 | 1 | 23993 | Klk7 | S | - |
| 12755 | 1 | 114294 | LACTB | 1 | 80907 | Lactb | - | U |
| 8670 | 1 | 27074 | LAMP3 | 1 | 239739 | Lamp3 | - | U |
| 55469 | 1 | 3949 | LDLR | 1 | 16835 | Ldlr | - | U |
| 38075 | 1 | 5641 | LGMN | 1 | 19141 | Lgmn | S | U |
| - | 1 | 732275 | LOC732275 | - | - | - | - | U |
| 34980 | 1 | 91694 | LONRF1 | 1 | 244421 | Lonrf1 | - | U |
| 20952 | 1 | 4036 | LRP2 | 1 | 14725 | Lrp2 | S | - |
| 1746 | 1 | 4041 | LRP5 | 1 | 16973 | Lrp5 | S | - |
| 37408 | 1 | 4047 | LSS | 1 | 16987 | Lss | S | - |
| 497 | 1 | 4049 | LTA | 1 | 16992 | Lta | - | U |
| 7406 | 1 | 4056 | LTC4S | 1 | 17001 | Ltc4s | - | U |
| 31085 | 1 | 4065 | LY75 | 1 | 17076 | Ly75 | - | U |
| 31315 | 1 | 9935 | MAFB | 1 | 16658 | Mafb | S | - |
| 8845 | 1 | 9794 | MAML1 | 1 | 103806 | Maml1 | - | U |
| 4316 | 1 | 4121 | MAN1A1 | 1 | 17155 | Man1a | - | U |
| 37670 | 1 | 5594 | MAPK1 | 1 | 26413 | Mapk1 | S | - |
| 31777 | 1 | 1432 | MAPK14 | 1 | 26416 | Mapk14 | S | - |
| 56412 | 1 | 9261 | MAPKAPK2 | 1 | 17164 | Mapkapk2 | - | U |
| 76766 | 1 | 10150 | MBNL2 | 1 | 105559 | Mbnl2 | - | U |
| 1789 | 1 | 4157 | MC1R | 1 | 17199 | Mc1r | S | - |
| 7413 | 1 | 4170 | MCL1 | 1 | 17210 | Mcl1 | - | U |
| 21002 | 1 | 5469 | MED1 | 1 | 19014 | Med1 | S | - |
| 68441 | 1 | 9968 | MED12 | 1 | 59024 | Med12 | S | - |
| 21067 | 1 | 9969 | MED13 | 1 | 327987 | Med13 | S | U |
| 22082 | 1 | 9282 | MED14 | 1 | 26896 | Med14 | S | - |
| 64602 | 1 | 10025 | MED16 | 1 | 216154 | Med16 | S | - |
| 3151 | 1 | 9440 | MED17 | 1 | 234959 | Med17 | S | - |
| 3552 | 1 | 9439 | MED23 | 1 | 70208 | Med23 | S | - |
| 40795 | 1 | 9862 | MED24 | 1 | 23989 | Med24 | S | - |
| 12329 | 1 | 90390 | MED30 | 1 | 69790 | Med30 | S | - |
| 8568 | 1 | 29079 | MED4 | 1 | 67381 | Med4 | S | - |
| 49674 | 1 | 2872 | MKNK2 | 1 | 17347 | Mknk2 | - | U |
| 208 | 1 | 4292 | MLH1 | 1 | 17350 | Mlh1 | S | - |
| 3659 | 1 | 4318 | MMP9 | 1 | 17395 | Mmp9 | S | - |
| 37620 | 1 | 4330 | MN1 | 1 | 433938 | Mn1 | S | - |
| 41871 | 1 | 64968 | MRPS6 | 1 | 121022 | Mrps6 | - | U |
| 210 | 1 | 4436 | MSH2 | 1 | 17685 | Msh2 | S | - |
| 31092 | 1 | 4609 | MYC | 1 | 17869 | Myc | S | - |
| 6098 | 1 | 55191 | NADSYN1 | 1 | 78914 | Nadsyn1 | S | - |
| 68418 | 1 | 9476 | NAPSA | 1 | 16541 | Napsa | - | U |
| 41035 | 1 | 728841 | NBPF8 | 0 | - | - | - | U |
| 30964 | 1 | 653361 | NCF1 | 1 | 17969 | Ncf1 | S | - |
| - | 1 | 654816 | NCF1B | - | - | - | - | U |
| - | 1 | 654817 | NCF1C | - | - | - | - | U |
| 374 | 1 | 4688 | NCF2 | 1 | 17970 | Ncf2 | S | - |
| 4764 | 1 | 8202 | NCOA3 | 1 | 17979 | Ncoa3 | - | U |
| 38052 | 1 | 8031 | NCOA4 | 2 | 27057 | Ncoa4 | S | - |
| 40920 | 1 | 23054 | NCOA6 | 1 | 56406 | Ncoa6 | S | - |
| 38166 | 1 | 9611 | NCOR1 | 1 | 20185 | Ncor1 | S | - |
| 31370 | 1 | 9612 | NCOR2 | 1 | 20602 | Ncor2 | S | - |
| 32336 | 1 | 4772 | NFATC1 | 1 | 18018 | Nfatc1 | S | - |
| 2971 | 1 | 4790 | NFKB1 | 1 | 18033 | Nfkb1 | S | - |
| 1873 | 1 | 4791 | NFKB2 | 1 | 18034 | Nfkb2 | - | U |
| 7863 | 1 | 4792 | NFKBIA | 1 | 18035 | Nfkbia | S | U |
| 10670 | 1 | 28512 | NKIRAS1 | 1 | 69721 | Nkiras1 | - | U |
| 88935 | 1 | 84166 | NLRC5 | 1 | 434341 | Nlrc5 | - | U |
| 3441 | 1 | 9111 | NMI | 1 | 64685 | Nmi | - | U |
| 11156 | 1 | 64127 | NOD2 | 1 | 257632 | Nod2 | S | - |
| 32049 | 1 | 4851 | NOTCH1 | 1 | 18128 | Notch1 | S | - |
| 89329 | 1 | 388677 | NOTCH2NL | 0 | - | - | - | U |
| 4498 | 1 | 4878 | NPPA | 1 | 230899 | Nppa | S | - |
| 3760 | 1 | 9971 | NR1H4 | 1 | 20186 | Nr1h4 | S | - |
| 40757 | 1 | 8856 | NR1I2 | 1 | 18171 | Nr1i2 | S | - |
| 3759 | 1 | 9970 | NR1I3 | 1 | 12355 | Nr1i3 | S | - |
| 45675 | 1 | 344901 | OSTN | 1 | 239790 | Ostn | S | - |
| 56419 | 1 | 5079 | PAX5 | 1 | 18507 | Pax5 | - | U |
| 7879 | 1 | 27250 | PDCD4 | 1 | 18569 | Pdcd4 | - | U |
| 1956 | 1 | 5150 | PDE7A | 1 | 18583 | Pde7a | - | U |
| 32055 | 1 | 5154 | PDGFA | 1 | 18590 | Pdgfa | S | - |
| 68454 | 1 | 2923 | PDIA3 | 1 | 14827 | Pdia3 | S | - |
| 37310 | 1 | 5251 | PHEX | 1 | 18675 | Phex | S | - |
| 20582 | 1 | 5287 | PIK3C2B | 1 | 240752 | Pik3c2b | - | U |
| 21249 | 1 | 5290 | PIK3CA | 1 | 18706 | Pik3ca | - | U |
| 100644 | 1 | 8395 | PIP5K1B | 1 | 18719 | Pip5k1b | - | U |
| 32059 | 1 | 5321 | PLA2G4A | 1 | 18783 | Pla2g4a | S | - |
| 11001 | 1 | 59338 | PLEKHA1 | 1 | 101476 | Plekha1 | - | U |
| 66630 | 1 | 23654 | PLXNB2 | 1 | 140570 | Plxnb2 | - | U |
| 385 | 1 | 5445 | PON2 | 1 | 330260 | Pon2 | - | U |
| 259 | 1 | 5449 | POU1F1 | 1 | 18736 | Pou1f1 | S | - |
| 21047 | 1 | 5465 | PPARA | 1 | 19013 | Ppara | S | - |
| 7485 | 1 | 10891 | PPARGC1A | 1 | 19017 | Ppargc1a | S | - |
| 56428 | 1 | 5494 | PPM1A | 1 | 19042 | Ppm1a | - | U |
| 4546 | 1 | 5504 | PPP1R2 | 1 | 66849 | Ppp1r2 | - | U |
| 6323 | 1 | 79706 | PRKRIP1 | 1 | 66801 | Prkrip1 | - | U |
| 7904 | 1 | 5621 | PRNP | 1 | 19122 | Prnp | - | U |
| 37680 | 1 | 5660 | PSAP | 1 | 19156 | Psap | - | U |
| 739 | 1 | 5732 | PTGER2 | 1 | 19217 | Ptger2 | S | - |
| 31000 | 1 | 5743 | PTGS2 | 1 | 19225 | Ptgs2 | S | - |
| 266 | 1 | 5741 | PTH | 1 | 19226 | Pth | S | - |
| 2113 | 1 | 5744 | PTHLH | 1 | 19227 | Pthlh | S | - |
| 2587 | 1 | 7803 | PTP4A1 | 1 | 19243 | Ptp4a1 | - | U |
| 7497 | 1 | 5771 | PTPN2 | 1 | 19255 | Ptpn2 | - | U |
| 2126 | 1 | 5788 | PTPRC | 1 | 19264 | Ptprc | - | U |
| 55699 | 1 | 5880 | RAC2 | 1 | 19354 | Rac2 | - | U |
| 48436 | 1 | 10635 | RAD51AP1 | 1 | 19362 | Rad51ap1 | - | U |
| 48145 | 1 | 5894 | RAF1 | 1 | 110157 | Raf1 | S | - |
| 20262 | 1 | 5914 | RARA | 1 | 19401 | Rara | S | - |
| 20263 | 1 | 5916 | RARG | 1 | 19411 | Rarg | S | - |
| 8078 | 1 | 64283 | RGNEF | 1 | 110596 | Rgnef | - | U |
| 2845 | 1 | 8787 | RGS9 | 1 | 19739 | Rgs9 | - | U |
| 37856 | 1 | 8767 | RIPK2 | 1 | 192656 | Ripk2 | - | U |
| 4102 | 1 | 6039 | RNASE6 | 1 | 78416 | Rnase6 | - | U |
| 55899 | 1 | 6093 | ROCK1 | 1 | 19877 | Rock1 | - | U |
| 10969 | 1 | 91543 | RSAD2 | 1 | 58185 | Rsad2 | - | U |
| 68389 | 1 | 860 | RUNX2 | 1 | 12393 | Runx2 | S | - |
| 2220 | 1 | 6256 | RXRA | 1 | 20181 | Rxra | S | - |
| 7923 | 1 | 6257 | RXRB | 1 | 20182 | Rxrb | S | - |
| 21373 | 1 | 6258 | RXRG | 1 | 20183 | Rxrg | S | - |
| 36150 | 1 | 795 | S100G | 1 | 12309 | S100g | S | - |
| 37716 | 1 | 6303 | SAT1 | 1 | 20229 | Sat1 | - | U |
| 90891 | 1 | 6307 | SC4MOL | 1 | 66234 | Sc4mol | - | U |
| 48353 | 1 | 950 | SCARB2 | 1 | 12492 | Scarb2 | - | U |
| 13349 | 1 | 55681 | SCYL2 | 1 | 213326 | Scyl2 | - | U |
| 27797 | 1 | 347735 | SERINC2 | 1 | 230779 | Serinc2 | - | U |
| 69399 | 1 | 1992 | SERPINB1 | 1 | 66222 | Serpinb1a | - | U |
| 74445 | 1 | 5271 | SERPINB8 | 1 | 20725 | Serpinb8 | - | U |
| 68070 | 1 | 5054 | SERPINE1 | 1 | 18787 | Serpine1 | S | - |
| 2266 | 1 | 6422 | SFRP1 | 1 | 20377 | Sfrp1 | S | - |
| 34525 | 1 | 113402 | SFT2D1 | 1 | 106489 | Sft2d1 | - | U |
| 48162 | 1 | 6504 | SLAMF1 | 1 | 27218 | Slamf1 | - | U |
| 20984 | 1 | 9120 | SLC16A6 | 1 | 104681 | Slc16a6 | - | U |
| 331 | 1 | 788 | SLC25A20 | 1 | 57279 | Slc25a20 | - | U |
| 41357 | 1 | 219855 | SLC37A2 | 1 | 56857 | Slc37a2 | - | U |
| 10711 | 1 | 57153 | SLC44A2 | 1 | 68682 | Slc44a2 | - | U |
| 31412 | 1 | 6526 | SLC5A3 | 1 | 53881 | Slc5a3 | - | U |
| 21197 | 1 | 4087 | SMAD2 | 1 | 17126 | Smad2 | S | - |
| 55937 | 1 | 4088 | SMAD3 | 1 | 17127 | Smad3 | S | - |
| 4363 | 1 | 6615 | SNAI1 | 1 | 20613 | Snai1 | S | - |
| 31127 | 1 | 6591 | SNAI2 | 1 | 20583 | Snai2 | S | - |
| 56557 | 1 | 22938 | SNW1 | 1 | 66354 | Snw1 | S | - |
| 2811 | 1 | 401548 | SNX30 | 1 | 209131 | Snx30 | - | U |
| 8276 | 1 | 6667 | SP1 | 1 | 20683 | Sp1 | S | - |
| 90788 | 1 | 6672 | SP100 | 1 | 20684 | Sp100 | - | U |
| 82192 | 1 | 3431 | SP110 | 1 | 109032 | Sp110 | - | U |
| 56031 | 1 | 11262 | SP140 | 1 | 434484 | Sp140 | - | U |
| 19482 | 1 | 221178 | SPATA13 | 1 | 219140 | Spata13 | - | U |
| 20156 | 1 | 6696 | SPP1 | 1 | 20750 | Spp1 | S | - |
| 2355 | 1 | 6713 | SQLE | 1 | 20775 | Sqle | - | U |
| 2043 | 1 | 5552 | SRGN | 1 | 19073 | Srgn | - | U |
| 37736 | 1 | 6717 | SRI | 1 | 109552 | Sri | - | U |
| 21428 | 1 | 6772 | STAT1 | 1 | 20846 | Stat1 | S | - |
| 2374 | 1 | 6781 | STC1 | 1 | 20855 | Stc1 | S | - |
| 2753 | 1 | 8614 | STC2 | 1 | 20856 | Stc2 | S | - |
| 47918 | 1 | 412 | STS | 1 | 20905 | Sts | - | U |
| 10313 | 1 | 55959 | SULF2 | 1 | 72043 | Sulf2 | - | U |
| 55918 | 1 | 6882 | TAF11 | 1 | 68776 | Taf11 | S | - |
| 55723 | 1 | 6874 | TAF4 | 1 | 228980 | Taf4a | S | - |
| 11768 | 1 | 6879 | TAF7 | 1 | 24074 | Taf7 | S | - |
| 2408 | 1 | 6929 | TCF3 | 1 | 21423 | Tcfe2a | S | - |
| 2407 | 1 | 6925 | TCF4 | 1 | 21413 | Tcf4 | S | - |
| 540 | 1 | 7040 | TGFB1 | 1 | 21803 | Tgfb1 | S | - |
| 3177 | 1 | 7046 | TGFBR1 | 1 | 21812 | Tgfbr1 | S | - |
| 31142 | 1 | 7057 | THBS1 | 1 | 21825 | Thbs1 | S | - |
| 31289 | 1 | 9967 | THRAP3 | 1 | 230753 | Thrap3 | S | - |
| 14161 | 1 | 92610 | TIFA | 1 | 211550 | Tifa | - | U |
| 12809 | 1 | 81793 | TLR10 | 0 | - | - | - | U |
| 20695 | 1 | 7097 | TLR2 | 1 | 24088 | Tlr2 | S | - |
| 41317 | 1 | 7099 | TLR4 | 1 | 21898 | Tlr4 | S | - |
| 49811 | 1 | 80008 | TMEM156 | 1 | 243025 | Tmem156 | - | U |
| 496 | 1 | 7124 | TNF | 1 | 21926 | Tnf | S | - |
| 4582 | 1 | 7128 | TNFAIP3 | 1 | 21929 | Tnfaip3 | S | - |
| 920 | 1 | 608 | TNFRSF17 | 1 | 21935 | Tnfrsf17 | - | U |
| 2744 | 1 | 8600 | TNFSF11 | 1 | 21943 | Tnfsf11 | S | - |
| 2495 | 1 | 7292 | TNFSF4 | 1 | 22164 | Tnfsf4 | S | U |
| 49589 | 1 | 7148 | TNXB | 1 | 81877 | Tnxb | S | - |
| 9447 | 1 | 10766 | TOB2 | 1 | 57259 | Tob2 | S | - |
| 460 | 1 | 7157 | TP53 | 1 | 22059 | Trp53 | S | - |
| 3960 | 1 | 7161 | TP73 | 1 | 22062 | Trp73 | S | - |
| 11885 | 1 | 80342 | TRAF3IP3 | 1 | 215243 | Traf3ip3 | - | U |
| 75216 | 1 | 10221 | TRIB1 | 1 | 211770 | Trib1 | - | U |
| 20830 | 1 | 8805 | TRIM24 | 1 | 21848 | Trim24 | S | - |
| 48427 | 1 | 10475 | TRIM38 | 1 | 214158 | Trim38 | - | U |
| 56812 | 1 | 55503 | TRPV6 | 1 | 64177 | Trpv6 | S | - |
| 9301 | 1 | 51061 | TXNDC11 | 1 | 106200 | Txndc11 | - | U |
| 38186 | 1 | 10628 | TXNIP | 1 | 56338 | Txnip | S | - |
| 834 | 1 | 7298 | TYMS | 1 | 22171 | Tyms | S | - |
| 84372 | 1 | 7324 | UBE2E1 | 1 | 22194 | Ube2e1 | - | U |
| 55739 | 1 | 7336 | UBE2V2 | 1 | 70620 | Ube2v2 | S | - |
| 23587 | 1 | 137886 | UBXN2B | 1 | 68053 | Ubxn2b | - | U |
| 121583 | 1 | 7366 | UGT2B15 | 2 | 243085 // 231396 | Ugt2b35 // Ugt2b36 | S | - |
| 84333 | 1 | 7367 | UGT2B17 | 3 | 112417 // 22238 // 100559 | Ugt2b37 // Ugt2b5 // Ugt2b38 | S | - |
| 57005 | 1 | 79805 | VASH2 | 1 | 226841 | Vash2 | - | U |
| 37297 | 1 | 7421 | VDR | 1 | 22337 | Vdr | S | U |
| 465 | 1 | 7428 | VHL | 1 | 22346 | Vhl | - | U |
| 22068 | 1 | 23230 | VPS13A | 1 | 271564 | Vps13a | - | U |
| 11649 | 1 | 79720 | VPS37B | 1 | 330192 | Vps37b | - | U |
| - | 1 | 339005 | WHAMML1 | - | - | - | - | U |
| 3963 | 1 | 7471 | WNT1 | 1 | 22408 | Wnt1 | S | - |
| 5894 | 1 | 54464 | XRN1 | 1 | 24127 | Xrn1 | - | U |
| 8563 | 1 | 29066 | ZC3H7A | 1 | 106205 | Zc3h7a | - | U |
| 8968 | 1 | 22882 | ZHX2 | 1 | 387609 | Zhx2 | - | U |
| 117700 | 1 | 10308 | ZNF267 | 0 | - | - | - | U |
| 8493 | 1 | 23036 | ZNF292 | 1 | 30046 | Zfp292 | - | U |
| 79457 | 1 | 163050 | ZNF564 | 1 | 619310 | Zfp872 | - | U |
| 65048 | 1 | 147949 | ZNF583 | 1 | 213011 | Zfp583 | - | U |
| 49448 | 1 | 51385 | ZNF589 | 0 | - | - | - | U |
| 83774 | 1 | 90592 | ZNF700 | 0 | - | - | - | U |

**Figure S1**, Heat maps of the 103 and 92 VDRGS-DLCGS overlapping genes in the DLCGS of C57BL6 mouse (A) and human (B) developing lung time series respectively. The expression signal of each gene in each time series has been standardized to average 0, variance 1 across their respective time intervals.

A.
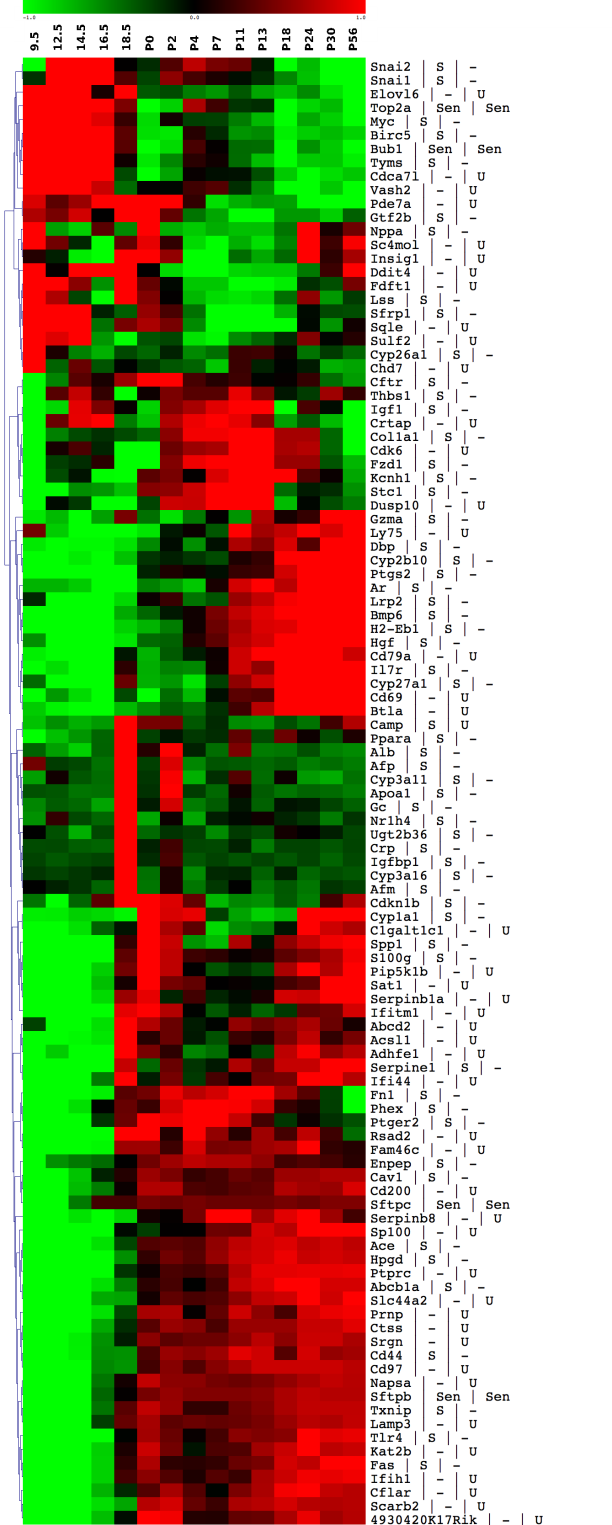
 B.
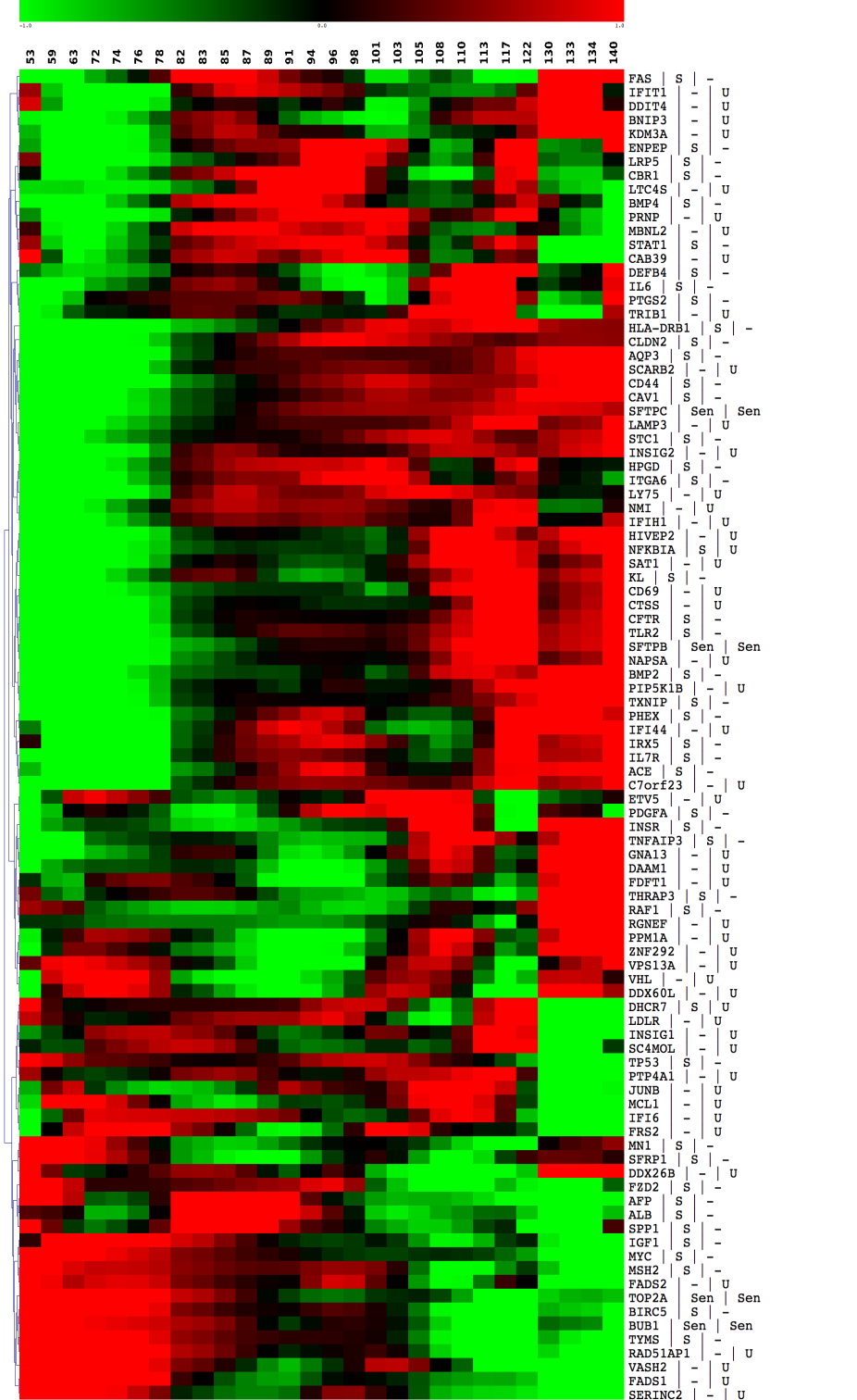

Supplement: Additional file 1: Table S1 — 413 vitamin D related genes. Figure S1. Heat maps of 103 and 92 vitamin D related lung genes overlapping with the developing lung characteristic genes of C57BL6 mouse (A) and human (B) developing lung time series respectively. The expression signal of each gene in each time series has been standardized to average 0, variance 1 across their respective time intervals. Four sentinel genes – BUB1, TOP2A, SFTPB and SFTPC – included for visual reference. [file 1755-8794-6-47-S1.doc]
